# Evaluation of the glottic surface dose in three‐dimensional conformal radiotherapy for early‐stage glottic cancer using a treatment planning system

**DOI:** 10.1002/acm2.70011

**Published:** 2025-04-14

**Authors:** Yuki Saito, Takahiro Kanehira, Takahito Yuki, Miyako Myojin

**Affiliations:** ^1^ Department of Radiation Oncology Keiyukai Sapporo Hospital Sapporo Japan; ^2^ Department of Medical Physics Hokkaido University Hospital Sapporo Japan

**Keywords:** collapsed cone convolution algorithm, Glottic cancer, photon Monte Carlo algorithm, Surface dose

## Abstract

**Background:**

In radiotherapy for early‐stage glottic cancer, evaluating the target surface dose at the glottic air–tissue boundary is crucial, as buildup effect can cause underdosing. The accuracy of dose evaluation in the surrounding tissues is affected by both the dose calculation algorithms and the accuracy of the Hounsfield unit values in the glottic air cavities.

**Purpose:**

The objective of this study is to investigate the impact of dose calculation algorithms and material override on glottic surface dose calculations in three‐dimensional conformal radiotherapy (3DCRT) for glottic cancer.

**Methods:**

We retrospectively included three patients with early‐stage glottic cancer treated with 3DCRT. Treatment planning based on the collapsed cone convolution (CCC) algorithm in the treatment planning system with a 1‐mm dose grid was conducted using a prescribed dose of 65 Gy in 26 fractions. The contours of the glottic air cavities and the surrounding glottic tissues were delineated for material override to air and water, respectively to assign correct materials in dose calculation. Each treatment plan was initially calculated using CCC without material override (CCC_w/o) and recalculated using CCC with material override (CCC_w) as well as photon Monte Carlo (pMC) algorithm with and without material override (pMC_w and pMC_w/o, respectively). A 1‐mm glottic surface dose (D_99%_) was evaluated using CCC_w/o, CCC_w, pMC_w, and pMC_w/o.

**Results:**

PMC_w predicted a ∼13.0% reduction in the glottic surface dose compared with the prescribed dose. CCC_w/o, CCC_w, and pMC_w/o overestimated the dose by ∼10.0% compared with pMC_w. The difference between CCC_w/o and pMC_w/o was minimal (0.6%); pMC_w/o significantly overestimated (by 10.8%) the dose compared with pMC_w, indicating the significant impact of material override in pMC.

**Conclusion:**

Monte Carlo dose calculations with material override are essential for the accurate surface dose calculation in 3DCRT for glottic cancer. Without appropriate material override, both CCC and pMC overestimate the surface dose.

## 1 INTRODUCTION

The dose buildup effect leads to a reduction in the surface dose at the air–tissue boundary.^1^, ^2^, ^3^, ^4^ In megavolt (MV) photon beam radiotherapy for early‐stage glottic cancer, this effect is a problem that cannot be ignored, as it can result in target surface underdosing, potentially compromising the treatment outcomes. Therefore, an accurate dose evaluation based on a treatment planning system (TPS) is essential to ensure the required dose.

The dose reduction at the air–tissue boundary using air cavity phantoms has been investigated in several studies, which demonstrated that the dose calculation accuracy depends on the dose calculation algorithms used.^5^, ^6^, ^7^, ^8^ The widely used convolution/superposition algorithms employed in TPSs, such as the collapsed cone convolution (CCC) and the analytical anisotropic algorithms, tend to overestimate the surface dose due to limitations in modeling secondary electron transport.^9^ In contrast, advanced dose calculation algorithms, such as Monte Carlo and Acuros XB (AXB) implemented in Eclipse TPS (Varian Medical Systems, Palo Alto, CA, USA), exhibited a good agreement with the experimental measurements. Despite these extensive studies, most previous investigations were limited to simplified phantoms. To the best of our knowledge, few studies have evaluated the surface dose in glottic cancer patients using computed tomography (CT) data,^10^ and the dose evaluation was performed in intensity‐modulated radiotherapy (IMRT). However, it remains largely uninvestigated in other beam delivery techniques including three‐dimensional conformal radiotherapy (3DCRT).

Moreover, inaccuracies in the Hounsfield unit (HU) values at the air–tissue boundary in CT images can further affect the dose evaluation. Cheung et al.^11^ reported significant discrepancies in the dose evaluation in volumetric modulated arc therapy for nasopharyngeal cancer, where air in the nasal cavity was incorrectly assigned as a lung tissue in the AXB algorithm. It has been reported that the HU values in air cavities within phantoms are higher than those in external air^12^; additionally, the HU values are incorrect at the air–issue boundary due to the partial volume effect, leading to inaccurate dose evaluation. Despite these studies, the impact of HU variation in the glottic air cavities on the surface dose evaluation for glottic cancer has yet to be investigated.

To address the above issues, in this study, we evaluate and compare the glottic surface dose in 3DCRT for glottic cancer using the CCC and photon Monte Carlo (pMC) dose calculation algorithms employed in RayStation (version 10A, RaySearch, Stockholm, Sweden) TPS. Additionally, we assess the impact of HU variation within the glottic air cavities on the surface dose evaluation by comparing the results obtained with and without material override. Furthermore, as reduction of the surface dose depended on field sizes and glottic cavities appear larger in the posterior part, we evaluated surface doses in different parts of glottis.

## 2 MATERIALS AND METHODS

### 2.1 Patient data and clinical target definition

We retrospectively included three patients with early‐stage glottic cancer who received radical radiotherapy with a prescribed 65 Gy dose in 26 fractions (2.5 Gy per fraction) using the Elekta Versa HD linear accelerator (Elekta AB, Stockholm, Sweden) located at our institution. All patients were scanned for treatment planning CT using an Aquilion Exceed LB CT scanner (Canon Medical Systems, Otawara, Japan), with a thermoplastic fixed shell (ESS‐25, ESFORM; Engineering System, Nagano, Japan), and gross tumor volumes were delineated on the planning CT images. In this study, the treatment planning CT images were reconstructed using a 1 mm × 1 mm × 1 mm voxel size to represent the accurate patient geometries.

### 2.2 Contours for material override

To assess the impact of the HU variations in the glottic air cavities on dose evaluation, we delineated the glottic air cavities for material override in the treatment planning CT. Since the visualization of the glottic air–tissue boundary varies with the window level/width setting, we determined the appropriate window level/width to visualize the actual boundary using the I'mRT Phantom (IBA Dosimetry GmbH, Schwarzenbruck, Germany), which contains known‐sized cylindrical air cavities according to the following procedure:

The I'mRT Phantom featuring two cylindrical air cavities with diameters of 7 and 13 mm, respectively, was scanned using our CT scanner, and the CT images were transferred to the RayStation TPS.

Two cylindrical regions of interest (ROIs) with diameters of 7 and 13 mm, respectively, were created and positioned at the center of each cavity identified on the CT images in the TPS.

The window level was adjusted to visually match the air cavities with the cylindrical ROIs on the CT images, and the window width was set to 0 for HU binarization.

**FIGURE 1:**
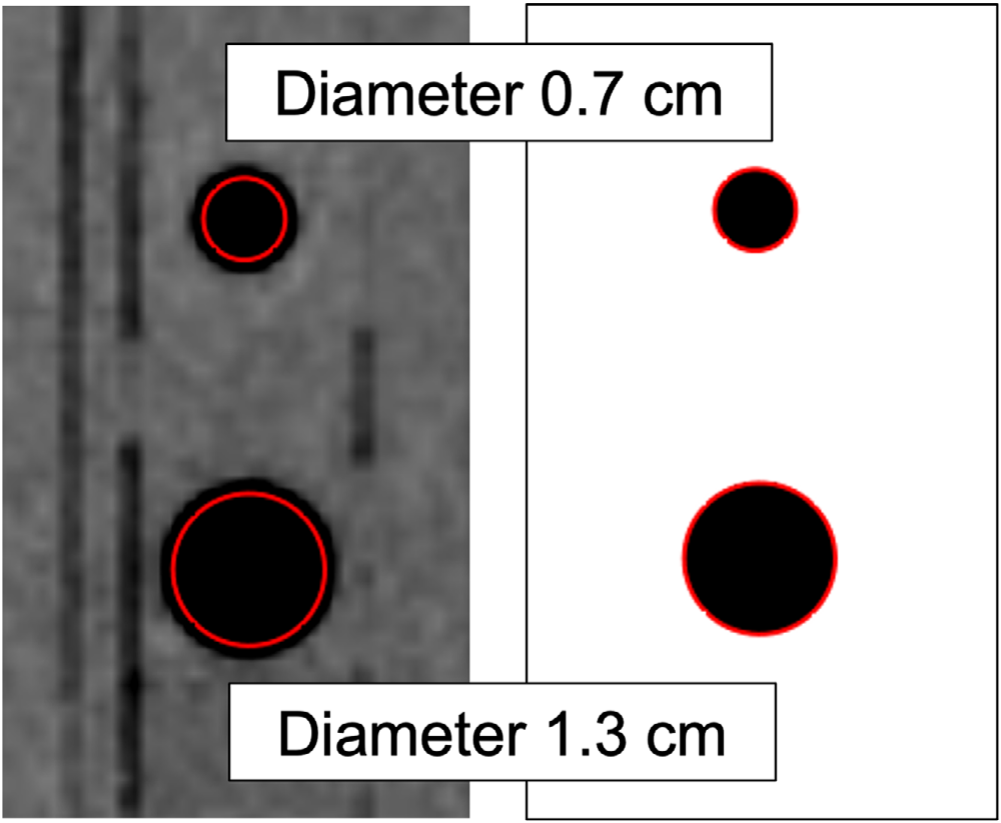
Axial CT images of the I'mRT Phantom with two air cavities with diameters of 0.7 cm and 1.3 cm displayed using two window level/width settings: (a) soft tissue (40 HU/350 HU); (b) determined setting for visualizing the actual air–tissue boundary (–500 HU/0 HU). The red circles with diameters corresponding to the 0.7 and 1.3 cm air cavities are displayed at the centers of the respective air cavities for reference. CT, computed tomography; HU, Hounsfield unit.

As a result, the window level was determined to be −500 HU. Figure 1 shows the CT images of the I'mRT Phantom with two air cavities displayed under the soft tissue level/width (40 HU/ 350 HU) and the determined level/width (−500 HU/0 HU).

**FIGURE 2:**
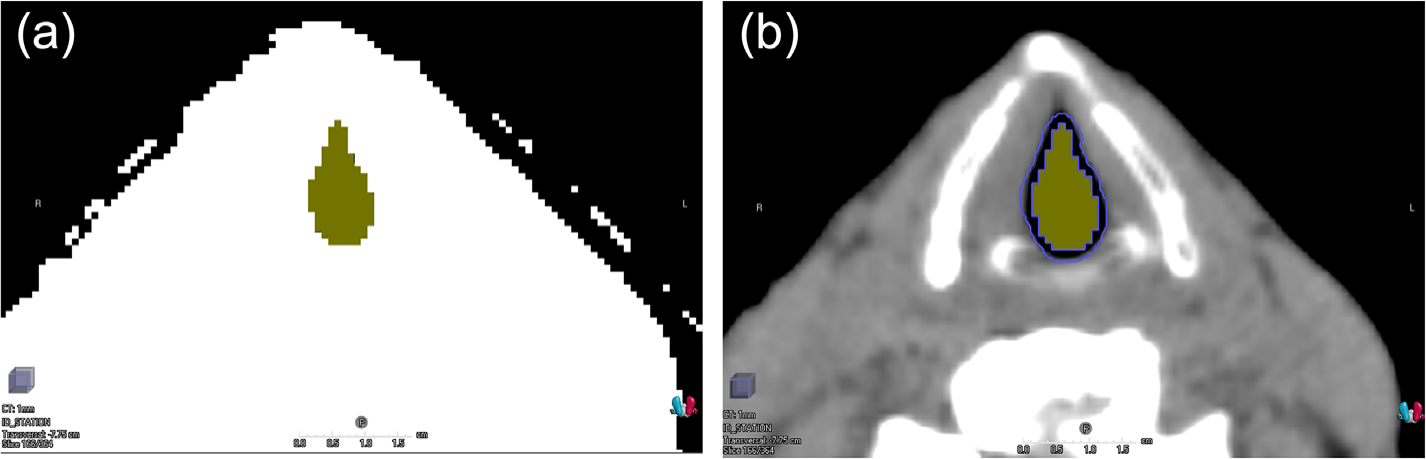
Axial CT images of the ROI delineation for material override in the glottic air cavity and surface displayed using two window level/width settings: (a) window level/width determined for the air–tissue boundary (–500 HU/0 HU); (b) soft tissue window level/width (40 HU/350 HU). The yellow wash represents the air ROIs of the glottic air cavity, and the blue contour represents the water ROIs of the glottic surface. CT, computed tomography; HU, Hounsfield unit; ROI, region of interest.

For material override in the glottic air cavities, air ROIs were delineated to include all pixels within the glottic air cavities identified below −500 HU (black region) in the window level/width (−500 HU/0 HU), as shown in Figure 2a, extending 5 mm in the superior–inferior direction from the isocenter of each treatment plan. Additionally, the region surrounding the air ROIs was considered to have a lower density than the actual glottic tissue density due to the partial volume effect. Thus, as shown in Figure 2b, water ROIs for material override of the glottic surface were generated as a 2‐mm thick ring expanded from the air ROIs in the anterior–posterior (AP) and left–right (LR) directions.

### 2.3 Treatment plans and dose calculation

For each patient, we generated 3DCRT plans using two beam arrangements: (1) two parallel opposed fields (gantry angles of 90° and 270°) and (2) three fields (gantry angles of 90°, 180°, and 270°). Three‐field plans were generated for actual treatment using the Pinnacle^3^ TPS (Philips Medical Systems, Fitchburg, WI, USA). A 4‐MV beam was used for gantry angles of 90° and 270°, and a 10‐MV beam was used for a 180° gantry angle. Wedges were applied at the 90° and 270° gantry angles. The beam weights were set to 42.5%, 15%, and 42.5% for the 90°, 180°, and 270° beams, respectively. The field size was set to 5–6 cm × 5.5 cm for each beam and adjusted to include the glottis investigated by oncologists (Figure SA1 and Table SA1). The isocenter was set approximately at the midpoint of the glottis, avoiding placement within the glottic air cavities. The treatment plans, structure sets, and 1‐mm voxel size treatment planning CTs were transferred to RayStation TPS. The dose for an isocenter was prescribed to 65 Gy in 26 fractions in the RayStation TPS with CCC using a 1‐mm dose grid size for all directions. Based on the three‐field plans, a two‐field plan with parallel opposed fields was generated by removing the 180° beams.

The dose in each plan was recalculated using three calculation settings: (1) CCC with material override (CCC_w) of the air and water ROIs to the air material (mass density: 0.00121 g/cm^3^) and water material (mass density: 1.000 g/cm^3^), respectively, (2) pMC without material override (pMC_w/o), and (3) pMC with material override (pMC_w). In total, for each plan, we generated four different dose distributions using CCC without material override (CCC_w/o), CCC_w, pMC_w/o, and pMC_w.

### 2.4 Dose evaluation

**FIGURE 3:**
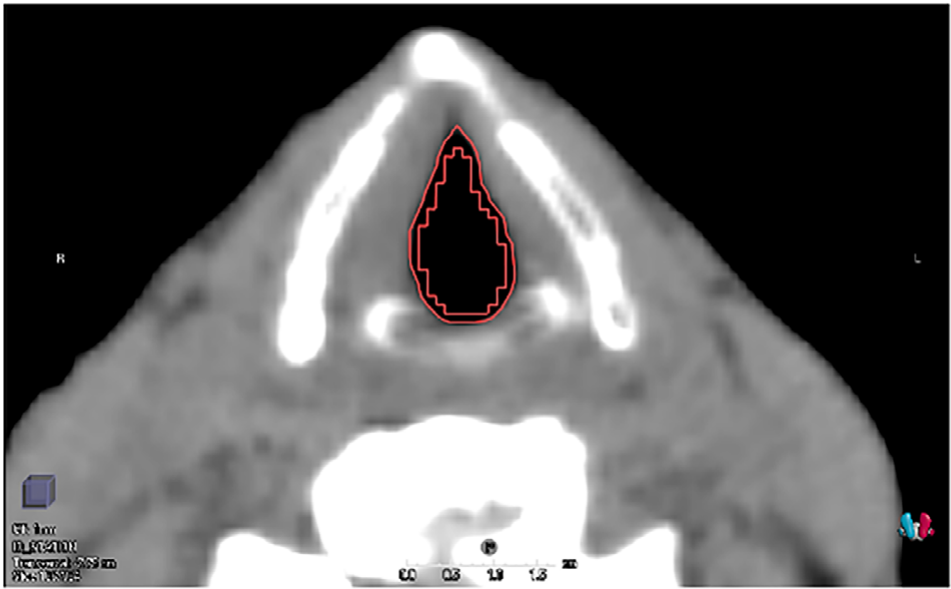
Axial CT images for delineation of surface ROIs. CT, computed tomography; ROI, region of interest.

To evaluate the glottic surface dose, surface ROIs were generated to include a 1‐mm margin surrounding the air ROIs in the AP and LR directions (Figure 3). The surface ROI doses were assessed for each treatment plan using D_99%_, which represents the near‐minimum dose covering 99% of the volume. The differences in D_99%_ for the surface ROIs relative to the prescribed dose were evaluated using CCC_w/o, pMC_w/o, CCC_w, and pMC_w. Here, we consider pMC_w as a reference dose calculation setting because of its highest accuracy. Additionally, to assess the effect of a dose calculation algorithm and material override on the glottic surface dose, we calculated the percentage differences in D_99%_ among the calculation settings. For example, the difference between pMC_w and CCC_w/o was computed as ([CCCw/o−pMC_w]/pMC_w)×100. The following comparisons were made: CCC_w/o against pMC_w, CCC_w/o against pMC_w/o, CCC_w/o against CCC_w, and pMC_w/o against pMC_w. Furthermore, we evaluated the surface dose reduction in different parts of the glottic surface relative to the prescribed dose using pMC_w (Figure SA2).

## 3 RESULTS

### 3.1 Dose distribution for four dose calculation settings

**FIGURE 4:**
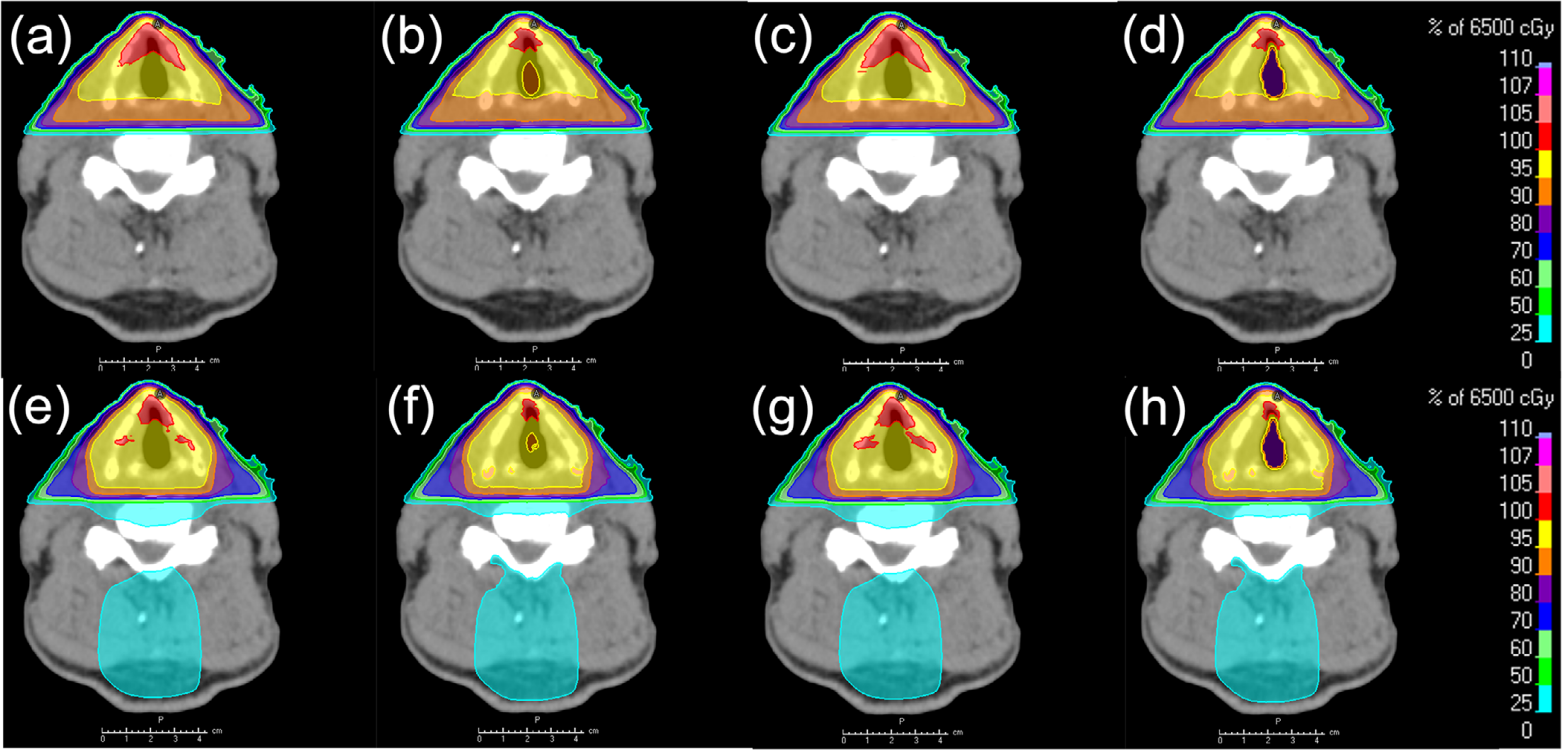
Dose distribution for glottic cancer with two‐field and three‐field plans for four calculation settings: (a) two‐field plan using CCC algorithm without material override, (b) two‐field plan using pMC algorithm without material override, (c) two‐field plan using CCC algorithm with material override, (d) two‐field plan using pMC algorithm with material override, (e) three‐field plan using CCC algorithm without material override, (f) three‐field plan using pMC algorithm without material override, (g) three‐field plan using CCC algorithm with material override, and (h) three‐field plan using pMC algorithm with material override. CCC, collapse cone convolution; pMC, photon Monte Carlo.

We performed dose calculations for the glottic surface dose using the CCC and pMC algorithms with and without material override settings to evaluate the impact of calculation algorithms and material correction. Figure 4 shows the dose distributions for two‐field and three‐field plans in the same patient, calculated with CCC_w/o, pMC_w/o, CCC_w, and pMC_w. In the CCC_w/o, pMC/w/o, and CCC_w calculations, dose reductions were not largely observed in the glottic air cavity and its surrounding tissue. In contrast, a dose reduction of more than 10% was observed in the pMC_w calculation.

### 3.2 Reduction in dose relative to the prescribed dose

**FIGURE 5:**
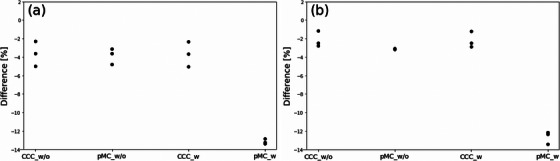
Point plots showing the relative differences in D_99%_ of the surface ROIs and the prescribed dose for each dose calculation setting (CCC_w/o, pMC_w/o, CCC_w, and pMC_w) for each patient using (a) two‐field plans and (b) three‐field plans. CCC_w, collapsed cone convolution with material override; CCC_w/o, collapsed cone convolution without material override; pMC_w, photon Monte Carlo with material override; pMC_w/o, photon Monte Carlo without material override; ROI, region of interest.

Figure 5 shows differences in D_99%_ of the surface ROIs relative to the prescribed 65 Gy dose for the dose calculation settings used. Using CCC_w/o, pMC_w/o, and CCC_w, the average D_99%_ values for the surface ROIs were 2%–4% lower than the prescribed dose. The two‐field plans reduced the average D_99%_ slightly more than the three‐field plans (–3.6% to –3.9% for the two‐field plans and –2.1% to –3.1% for the three‐field plans). In contrast to CCC_w/o, pMC_w/o, and CCC_w, the average D_99%_ values for pMC_w were 13.1% and 12.6% lower than the prescribed dose for two‐ and three‐field plans, respectively.

### 3.3 Impact of dose calculation algorithms and material override on the glottic surface dose

**FIGURE 6:**
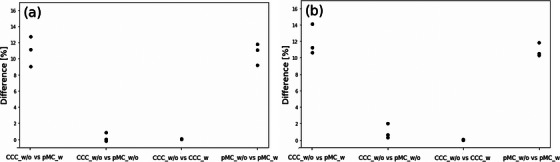
Point plots of the percentage differences in D_99%_ of the surface ROIs (CCC_w/o against pMC_w, CCC_w/o against pMC_w/o, CCC_w/o against CCC_w, and pMC_w/o against pMC_w) for each patient for (a) two‐field plans and (b) three‐field plans. CCC_w, collapsed cone convolution with material override; CCC_w/o, collapsed cone convolution without material override; pMC_w, photon Monte Carlo with material override; pMC_w/o, photon Monte Carlo without material override; ROI, region of interest.

Figure 6 shows a comparison of the D_99%_ values for the surface ROIs calculated between dose calculation settings.

A comparison between the clinical dose calculation setting (CCC_w/o) and our reference (pMC_w) showed that the average D_99%_ values for CCC_w/o were 11.0% and 12.0% larger than those for pMC_w for the two‐field and three‐field plans, respectively.

In the assessment of the impact of different dose calculation algorithms on the glottic surface dose without material override, the differences in the average D_99%_ values between pMC_w/o and CCC_w/o were 0.2% and 1.0% for the two‐field and three‐field plans, respectively.

Regarding the impact of material override on the glottic surface dose for each dose calculation algorithm, the differences in the average D_99%_ values between CCC_w and CCC_w/o were 0.05% and 0.04% for the two‐field and three‐field plans, respectively. On the other hand, the average D_99%_ values for pMC_w/o were 10.7% and 10.9% larger than those for pMC_w for the two‐field and three‐field plans, respectively.

The surface doses in the anterior, middle, and posterior parts of the glottis using pMC_w were shown in Figure SA2. The average reduction in the D_99%_ value from the prescribed dose increased toward the posterior for the two‐fields plans, which was not observed for the three‐field plans (Figure SA3).

## 4 DISCUSSION

We evaluated the glottic surface dose in 3DCRT for glottic cancer using the CCC and pMC algorithms in TPSs by evaluating the effect of correcting the material assigned in the glottic air cavities and the surrounding tissues. The results showed a substantial 13.0% reduction in the glottic surface dose compared with the prescribed dose when using the reference dose pMC calculation with material override; a 2%–4% reduction was observed for other dose calculation settings. Compared with pMC_w, CCC and pMC_w/o predicted a 10% higher glottic surface dose, indicating the limitations of both the clinically widely used superposition/convolution algorithm and pMC_w/o. The 0.6% dose difference observed between CCC_w/o and pMC_w/o is small compared with the large 10.8% difference observed between pMC_w and pMC_w/o, indicating the substantial impact of proper material override when using the pMC algorithm. These results show that the accurate dose required for the glottic surface must be evaluated using advanced dose calculation algorithms such as Monte Carlo algorithm with proper material assignments within the glottic air cavities and the surrounding tissues.

The 13% dose reduction observed using pMC_w is consistent with previous simplified phantom studies, where a dose reduction at the air–tissue boundary was reported; however, the magnitude of reduction varied, ranging up to 56%, depending on factors such as field size, beam energy, and geometry.^13^, ^14^ In our study, the difference between CCC and pMC_w/o was only 0.5%, which is consistent with the small discrepancies (< 2%) observed between CCC and AXB in IMRT for glottic cancer.^10^ However, when material override was applied to the glottic air cavities and the surrounding tissues, the dose discrepancies between CCC and pMC were increased to ∼10%, indicating the importance of considering the air cavities and the tissue density variations in accurate dose evaluations. These results in 3DCRT show that using material override can also be critical in the IMRT settings.

**FIGURE 7:**
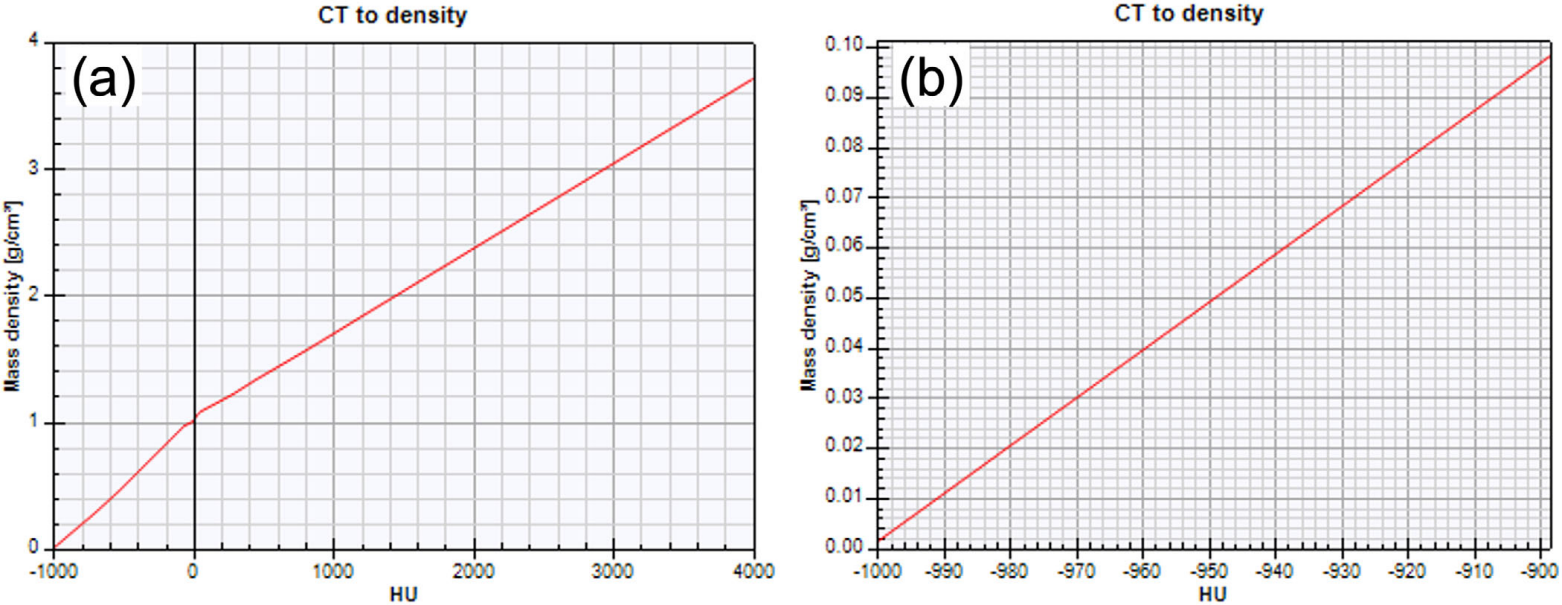
CT‐to‐mass density table used in our institution: (a) full range (from −1000 to 4000 HU); (b) low‐density range (from −1000 to −900 HU). CT, computed tomography; HU, Hounsfield unit.

Although the Monte Carlo dose calculation is considered the most accurate in radiotherapy, our results showed a 10.8% overestimation of the glottic surface dose without material override. This discrepancy is probably due to the increased CT values in the glottic air cavities compared with the actual air (−1000 HU), which was also reported in a previous study where the CT values for air inside a phantom were found to be higher than those for external air.^12^ Additionally, the CT values in the glottic air at the air–tissue boundary were increased due to the partial volume effect. These increased CT values resulted in the assignment of a higher density in the glottic air cavity according to the CT‐to‐mass density table shown in Figure 7. Figure 7a shows the full range (from −1000 to 4000 HU), and Figure 7b shows the table covering the low‐density range (from −1000 to −900 HU.) As shown in Figure 7b, a mere 10‐HU change in the CT value (from −1000 to −990 HU) results in a nearly ninefold increase in the mass density (from 0.00121 to ∼0.01 g/cm^3^), which can affect the pMC simulation of secondary electrons at the air–tissue boundary. Consequently, to improve the accuracy of the glottic surface dose calculations, it is essential to assign the correct material and density when using the pMC algorithm.

This uncertainty in CT values affecting the accuracy of dose calculation was corrected by manual density override in this study. Dual‐energy CT might address this issue by providing more accurate CT values compared to the widely used single‐energy CT,^15^ making it an area of interest for future studies.

Figure 1a shows that in the soft tissue level/range (40 HU/350 HU), the air cavity diameter appears to be ∼1.5 mm larger than the actual diameter. Therefore, careful consideration is needed when contouring targets and air regions to evaluate the surface dose for tumors close to the air cavity of the glottis in the CT images of patients. The air threshold value used, which was based on the I'mRT Phantom CT image was −500 HU. However, this threshold may vary depending on patient‐specific data and different CT scanners manufacturers.

Regarding treatment outcomes, the importance of fraction size in early‐stage glottic cancer has been discussed in several studies. Yamazaki et al.^16^ reported that in T1 glottic cancer, the 5‐year local control rates for 2.0 and 2.25 Gy fraction sizes were 77% and 92%, respectively, indicating significantly improved results when using a high fraction size. Similarly, Le et al.^17^ found that for T1–T2 glottic cancer, a 2.25 Gy/fraction resulted in a significantly higher 5‐year local control rate than that using a 1.8 Gy/fraction. Furthermore, Yu et al.^18^ concluded that the most critical factor that determines local control in glottic cancer is the fraction size. In our study, D_99%_ in the surface ROI was decreased by ∼13.0% compared with that of the prescribed dose, which could lead to a minimum surface dose of ∼1.74 Gy/fraction for a 2.0 Gy fraction size, potentially resulting in poor local control (given typically 2 Gy/fraction required for head and neck tumors). However, when using a 2.25 Gy fraction size, a sufficient fraction dose of almost 2.0 Gy could be maintained despite the dose reduction caused by the dose buildup effect, possibly contributing to improved local control.

**FIGURE 8:**
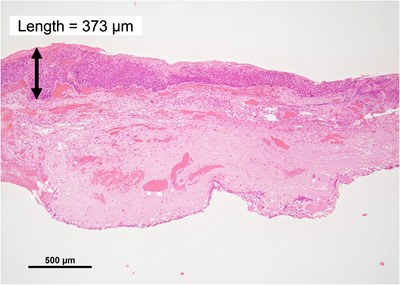
Pathological image of glottic cancer obtained in our institution. “Length” indicates that the cancer cells are located at 373 µm below the glottic surface.

This study has the following limitations: (1) We calculated the dose, which closely approximates the actual dose, using pMC_w as a reference dose calculation but did not compare it with the dose measured using an ion chamber or a film; nevertheless, our pMC evaluation met the dosimetric tolerance reported in the ESTRO Booklet No. 7^19^ and was validated with film measurements in an inhomogeneous phantom with an air layer, as reported in our previous studies,^7^, ^8^ which also exhibited better agreement with the dose measured using the film than the dose calculated using CCC. (2) We included only three patients; this limits the generalizability of the results and cannot reflect interpatient variability. (3) We used a 1‐mm resolution calculation grid, which is the finest grid available in RayStation TPS; however, as shown in Figure 8, glottic cancer cells are located at the depth of less than 1 mm from the glottic surface; therefore, the actual dose delivered to these cells may be even lower than the calculated dose.

## 5 CONCLUSION

In this study, we investigated the glottic surface dose in 3DCRT for an early‐stage glottic cancer by comparing the dose evaluated using CCC_w, CCC_w/o, pMC_w, and pMC_w/o. The results showed a substantial 13.0% dose reduction compared with the prescribed dose for the glottic surface when using pMC_w. CCC_w/o, CCC_w and even pMC_w/o overestimated the surface dose by more than 10% compared with pMC_w. These findings demonstrate the importance of using pMC with appropriate material override for accurate doses evaluation in glottic cancer.

## AUTHOR CONTRIBUTIONS

Yuki Saito, Miyako Myojin, and Takahito Yuki collected the data and prepared the materials. Yuki Saito, Miyako Myojin, Takahito Yuki, and Takahiro Kanehira contributed to the study conception and design. Yuki Saito, Miyako Myojin, Takahito Yuki, and Takahiro Kanehira participated in data analysis. Yuki Saito and Takahiro Kanehira wrote the first draft of the manuscript. All authors commented on the manuscript and approved the final manuscript.

## CONFLICT OF INTEREST STATEMENT

The authors declare no conflicts of interest.

## Supporting information



Supporting Information
