# Supplementary material for: Evaluation of the glottic surface dose in three‐dimensional conformal radiotherapy for early‐stage glottic cancer using a treatment planning system
Source: J Appl Clin Med Phys. 2025 Apr 14;26(5):e70011. doi: 10.1002/acm2.70011 (PMC12059279; doi:10.1002/acm2.70011)
Supplement: Supplementary file 1 — Supporting Information [file ACM2-26-e70011-s001.docx]

**Appendix A: Field shape and size details**

For each patient, we generated treatment plans for 3DCRT with two parallel opposed fields (gantry angles of 90^o^ and 270^o^), and three fields (gantry angles of 90^o^, 180^o^, and 270^o^). Appendix-Fig. 1(a) shows beam’s eye view (BEV) for 90^o^, and Appendix-Fig. 1(b) shows BEV for 180^o^. The shapes and sizes of irradiation field were adjusted from a 5–6 cm × 5.5 cm square field to include glottis by oncologists. Appendix-Table 1 shows the field sizes for each beam angle for each patient.

Appendix-Fig. 1. Beam’s eye view for treatment plan of glottic cancer: (a) 90^o^ beam, and (b) 180^o^ beam. Red color wash represents surface ROI (ROI: region of interest).

Appendix-Table 1. Field size with each beam angle for each patient.

| Patient | Beam angle | | |
| --- | --- | --- | --- |
|  | 90^o^ | 180^o^ | 270^o^ |
| 1 | 5.5×6.0 | 5.5×6.0 | 5.5×6.0 |
| 2 | 5.5×6.6 | 5.5×6.6 | 5.5×6.6 |
| 3 | 6.0×6.5 | 6.0×6.5 | 6.0×6.5 |

**Appendix B: Dose evaluation in anterior, middle and posterior glottis**

Several studies have demonstrated that dose reduction becomes more pronounced with larger air gaps [20-22]. In the case of the air cavity in the glottis, the width is greater in the posterior than in the anterior. To evaluate the glottic surface dose in anterior and posterior regions, we equally divided the surface ROIs into three regions along anterior-posterior direction: anterior (S_Ant), middle (S_Mid), and posterior (S_Post), as shown in Appendix-Fig. 2. Appendix-Fig. 3 presents the relative differences in the D_99%_ of the S_Ant, S_Mid and S_Post to the prescribed dose using photon Monte Carlo with material override (pMC_w). In the two-field plans, the average difference in the D_99%_ for the S_Ant, S_Mid and S_Post were -12.4%, -13.2%, and -13.6%, respectively. The surface doses were shown to be slightly lower toward the posterior glottis in two-field plans. Considering that the posterior glottic cavity appears to be larger, these results agree with previous investigations showing greater dose reduction in larger air gap. In contrast, in the three-field plans, the average differences in the D_99%_ for the S_Ant, S_Mid and S_Post were -12.4%, -12.9%, and -12.5%, respectively. The trend of greater dose reduction in the posterior part observed in the two-field plans was not observed in three-fields plans. This could be because the dose reductions caused by the two fields were mitigated by the additional beam in three fields.

Appendix-Fig. 2. Axial CT images for delineation of surface ROIs divided into three regions: S_Ant shown in green, (S_Mid) shown in yellow, and S_Post shown in orange (ROI: region of interest; S_Ant: surface anterior ROI; S_Mid: surface middle ROI; S_Post: surface posterior ROI).

Appendix-Fig. 3. Point plots of the relative difference in D_99%_ of S_Ant, S_Mid, and S_Post and the prescribed dose evaluated using pMC_w for each patient for (a) two-field plans and (b) three-field plans (CCC_w/o: collapsed cone convolution without material override; pMC_w: photon Monte Carlo with material override; ROI: region of interest; S_Ant: surface anterior ROI; S_Mid: surface middle ROI; S_Post: surface posterior ROI).
